# Neoadjuvant and adjuvant chemotherapy with doxorubicin and ifosfamide for bone sarcomas in adult and older patients

**DOI:** 10.3892/ol.2014.2567

**Published:** 2014-09-26

**Authors:** HIROSHI URAKAWA, SATOSHI TSUKUSHI, HIDESHI SUGIURA, KENJI YAMADA, YOSHIHISA YAMADA, EIJI KOZAWA, EISUKE ARAI, NAOHISA FUTAMURA, NAOKI ISHIGURO, YOSHIHIRO NISHIDA

**Affiliations:** 1 Department of Orthopaedic Surgery, Nagoya University Graduate School and School of Medicine, Nagoya, Aichi 466-8550, Japan; 2 Department of Orthopedic Surgery, Aichi Cancer Center Hospital, Nagoya, Aichi 464-8681, Japan; 3 Orthopedic Surgery, Aichi Cancer Center Aichi Hospital, Okazaki, Aichi 444-0011, Japan; 4 Orthopedic Surgery, Nagoya Memorial Hospital, Nagoya, Aichi 468-8520, Japan

**Keywords:** doxorubicin, ifosfamide, chemotherapy, bone sarcoma

## Abstract

The present study investigated the safety and efficacy of neoadjuvant and adjuvant chemotherapy with doxorubicin and ifosfamide for bone sarcoma in adult and older patients. A total of 18 consecutive patients with bone sarcoma (American Joint Committee on Cancer stage II in 14 patients and stage IV in four) treated with neoadjuvant and adjuvant chemotherapy at Nagoya Musculoskeletal Oncology Group hospitals in Japan between 2004 and 2011 were reviewed. The treatment efficacy and side-effects were evaluated. The responses to neoadjuvant chemotherapy were stable disease in 11 patients and progressive disease in three. Among the 12 evaluable patients, there were five with ≥90% tumor necrosis. The estimated overall survival (OS) rate at five years for the patients without metastasis prior to treatment was 56%. Major grade 3 or 4 side-effects included leukopenia in 14 cases, anemia in seven, thrombocytopenia in three, nausea in two and febrile neutropenia in two. One patient discontinued chemotherapy due to a temporarily depressed level of consciousness with arrhythmia (grade 2). The estimated five-year OS rate in this study was acceptable in patients without metastasis prior to treatment. A better coordinated prospective study of this combination regimen for older patients with bone sarcoma will be required to clarify its efficacy and tolerability.

## Introduction

There are two peaks of osteosarcoma incidence, as determined by population-based surveillance (1). The first is during adolescence, and the later peak is in the eighth decade. Neoadjuvant and adjuvant chemotherapy protocols have improved long-term survival, particularly in young patients with osteosarcoma. In population-based data, the relative five-year survival rates are 61.6, 58.7, and 24.2% in osteosarcoma patients aged 0–24, 25–59 and 60–85 years, respectively (1), with older patients having a poorer prognosis. Malignant fibrous histiocytoma (MFH) and dedifferentiated chondrosarcoma are known to occur in elderly patients (2,3).

Phase II studies of single agents and combinations of drugs have documented the efficacy of methotrexate (MTX), doxorubicin (DXR) and cisplatin (CDDP) in advanced osteosarcoma (4–6), leading to randomized studies confirming the efficacy of adjuvant chemotherapy (7,8). This three-drug combination represents the standard care for osteosarcoma. Ifosfamide (IFM), in studies with other agents, has been shown to exhibit significant activity in recurrent or metastatic osteosarcoma (9,10). However, IFM did not improve the degree of tumor necrosis in a study of patients with osteosarcoma who were treated with a neoadjuvant MTX, DXR, and CDDP regimen, with or without IFM (11).

Neoadjuvant and adjuvant chemotherapy protocols have not been established specifically for adult and older patients with bone sarcomas. Furthermore, the completion rate of regimens that have included CDDP and/or MTX has been reported to be low in this population (12,13). High completion rates of neoadjuvant and adjuvant chemotherapy with DXR and IFM have been reported in older patients with soft-tissue sarcoma (14,15). The present study was undertaken to determine the safety and efficacy of neoadjuvant and adjuvant chemotherapy with DXR and IFM for bone sarcoma in adult and older patients.

## Patients and methods

### Patients

Patients with bone sarcoma treated with neoadjuvant and adjuvant chemotherapy in four Nagoya Musculoskeletal Oncology Group Hospitals (Nagoya University Hospital, Aichi Cancer Center Hospital, Aichi Cancer Center Aichi Hospital and Nagoya Memorial Hospital) in Japan between January 2004 and February 2011 were reviewed. Patients aged >40 years with osteosarcoma and MFH of the bone were prospectively treated with IFM+DXR in these institutions. Other bone sarcomas in patients of all ages were treated with IFM+DXR according to the attending physician’s decision. After obtaining a waiver of patient informed consent requirements from the institutional review board, 18 consecutive patients with bone sarcoma were retrospectively reviewed. This study was approved by the ethics committee of Nagoya University Graduate School and School of Medicine (Nagoya, Japan).

### Patient characteristics and treatment regimens

Baseline demographic and clinical characteristics are listed in Table I. The study group consisted of 12 males and six females, with a median age of 63 years (range, 28–76 years). Histological subtypes were osteosarcoma (n=10), MFH of the bone (n=4), dedifferentiated chondrosarcoma (n=3) and angiosarcoma of the bone (n=1). According to the staging system of the American Joint Committee on Cancer (16), there were four patients of stage IIA, ten of stage IIB, one of stage IVA and three of stage IVB. The timing of chemotherapy was neoadjuvant and adjuvant in seven patients, neoadjuvant only in seven and adjuvant only in four. The median number of cycles of chemotherapy was 4 (range, 1–7). Patients were treated with neoadjuvant and/or adjuvant chemotherapy with 50–60 mg/m^2^ DXR and 6–10 g/m^2^ IFM every 3–4 weeks.

### Treatment analysis

Side-effects were graded according to the Common Terminology Criteria for Adverse Events v4.0 (17). The treatment efficacies were evaluated according to Response Evaluation Criteria In Solid Tumors (RECIST) v1.1 analysis of magnetic resonance imaging (18), necrosis rate of resected tumor and patient outcome.

### Statistical analysis

The overall survival (OS) rate from definitive treatment was calculated using Kaplan-Meier product limit methods. A log-rank test was used to identify differences in survival rates between groups. P<0.05 was used to indicate a statistically significant difference.

## Results

### Definitive treatments

One patient with metastasis could not undergo definitive surgery due to disease progression during neoadjuvant chemotherapy. The sites of definitive treatment for the primary tumors were the femur (n=4), tibia (n=3), humerus (n=2), pelvis (n=2), sternum (n=2) and spine (n=1) in patients without metastasis. The metastatic sites of definitive treatment for patients with metastatic tumors were the lung only (n=1), the lymph nodes only (n=1), and the bone and soft tissues (n=1). Definitive treatments were surgery in 15 cases, heavy ion radiation in one case and surgery and heavy ion radiation in one case each. Sites of definitive treatment were primary of the extremity in nine cases, primary of the trunk in five, primary and metastasis in two, and solitary distant recurrence in one.

### Treatment response

**Figure 1:**
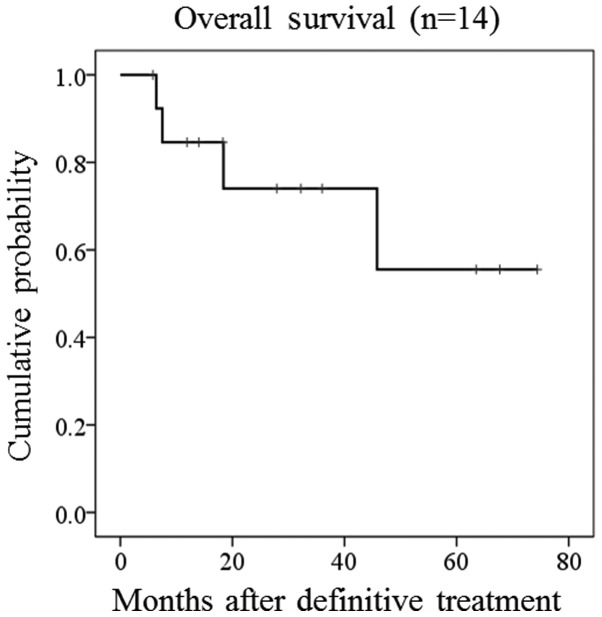
Kaplan-Meier estimated overall survival for 14 osteosarcoma patients without metastasis.

**Figure 2:**
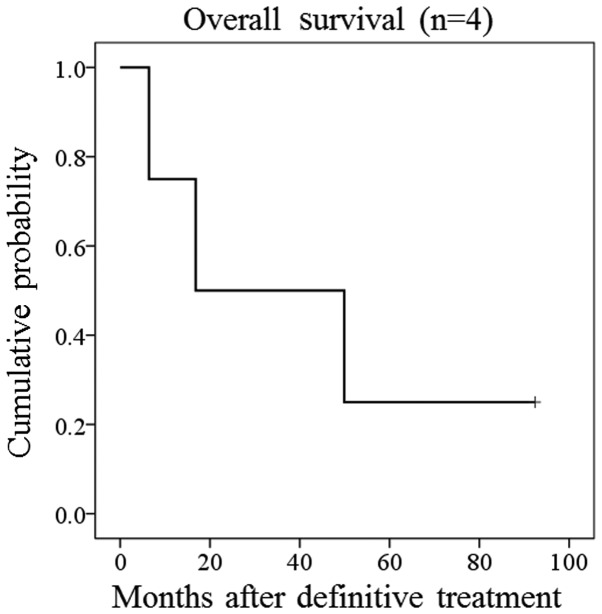
Kaplan-Meier estimated overall survival for four osteosarcoma patients with metastasis.

**Figure 3:**
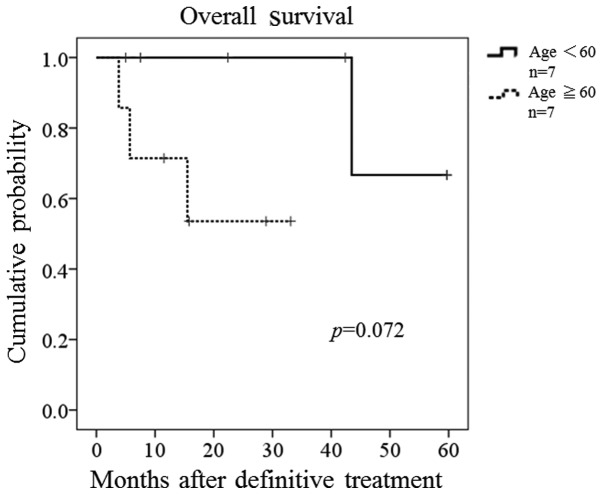
Kaplan-Meier estimated overall survival for seven osteosarcoma patients aged <60 years and seven patients aged >60 years without metastasis. There was no statistical difference between the two groups (P=0.072).

Evaluation with RECIST for the treatment site showed that the responses following neoadjuvant chemotherapy, with a median number cycles of 3.5 (range, 1–5), were stable disease (SD) in 11 cases (79%) and progressive disease (PD) in three (21%) (Table II). Of the 12 patients with SD and PD, there were five (42%) with tumor necrosis of ≥90% (Table II). The final status was no evidence of disease in nine cases (50%), alive without disease in two (11%) and dead of disease in seven (39%), at a median follow-up time of 18.0 months (range, 4.6–73.7 months) (Table II). Estimated five-year OS rates for patients without and with metastasis prior to treatment were 56% (Fig. 1) and 25% (Fig. 2), respectively (Table II). Estimated two-year OS rates for patients without metastasis were 100% in patients aged <60 years, and 54% in patients >60 years (P=0.072 between the two groups) (Fig. 3).

One patient discontinued chemotherapy due to a temporarily depressed level of consciousness with arrhythmia (grade 2). Major grade 3 or 4 side-effects included leukopenia in 14 cases (78%), anemia in seven (39%), thrombocytopenia in three (17%), nausea in two (11%), and febrile neutropenia in two (11%). Cardiotoxicity was not observed in the follow-up periods.

## Discussion

The number of aged osteosarcoma patients is increasing (19,20). Chemotherapy with DXR, CDDP and high-dose MTX has become a standard neoadjuvant and adjuvant treatment for osteosarcoma of young patients. However, older patients have been reported to receive less chemotherapy and have a poorer outcome (19). Since the completion rate of regimens, including CDDP and/or MTX, has been reported to be low in middle-aged and older bone sarcoma patients (12,13), a chemotherapeutic regimen with DXR and IFM was prospectively applied to adult and older patients with bone sarcomas in the present study. This regimen has already been widely used for adult patients with soft-tissue sarcoma (15,21). In the present study, only one patient discontinued chemotherapy due to a temporarily depressed level of consciousness with arrhythmia, suggesting that the combination of DXR and IFM is well tolerated, even in patients >60 years (22).

High-dose IFM treatment was reported to be associated with a higher incidence of azoospermia (23). However, this is less of a disadvantage with respect to infertility in the case of older patients. DXR-induced cardiotoxicity has been reported in the long-term follow-up of osteosarcoma (24); however no cardiotoxicity was noted in the present study, albeit within only a short median follow-up period of 18.0 months (range, 4.6–73.7 months).

Bramwell *et al* reported that adjuvant and/or neoadjuvant chemotherapy is beneficial in patients with MFH of the bone (13). The merit of adjuvant and/or neoadjuvant chemotherapy for dedifferentiated chondrosarcoma has not been proven (3). However, the present study performed adjuvant chemotherapy with IFM and DXR due to the poor outcome in patients with the sarcoma.

Previous studies have demonstrated ≥90% tumor necrosis in 21–48% of cases following neoadjuvant chemotherapy in osteosarcoma patients aged >40 years (12,19,25,26). In a study on MFH of the bone, ≥90% tumor necrosis was observed in 42% of patients subsequent to chemotherapy (13). In the present study, the responses following neoadjuvant chemotherapy were SD in 11 cases and PD in three. Five of 12 patients (42%) with bone sarcoma had ≥90% necrosis, and the efficacy of this treatment was acceptable.

In studies on neoadjuvant and/or adjuvant chemotherapy for non-metastatic osteosarcoma of an extremity, the estimated five-year OS rate was reported as 55–70% in patients aged 40–60 years (12,25) and 51% in patients >40 years (19). In MFH of the bone, the five-year OS rate following neoadjuvant and/or adjuvant chemotherapy was 59% in non-metastatic patients with a median age of 42 years (range, 14–62 years) (13). In non-metastatic patients aged <60 years who underwent limb salvage for dedifferentiated chondrosarcoma, the five-year OS rates were reported as 25% in those who did not have chemotherapy compared with 45% in those who did. However, no significant difference was noted between these groups on univariate analysis (3). In the present study, even though there were five tumors located in the axial skeleton and nine in the extremities, and the median age of the patients was 63 years (range, 28–76 years), the five-year OS rate of 56% was comparable to that of previous reports regarding bone sarcoma in patients without metastasis (3,12,13,19,25).

There were certain limitations to the present study. The first was the diversity of the chemotherapy dose and cycles based on the attending physician’s decision. A combination regimen with DXR (60 mg/m^2^) and IFM (10 g/m^2^) was typically selected, used in four cycles prior to surgery and two cycles subsequent to surgery, but certain cases were treated with different doses and cycles according to the attending physician’s decision. The second limitation was the short follow-up periods in this study. In particular, the follow-up periods in the older patients were short, and did not allow calculation of the estimated OS rate at five years in this population. Furthermore, the study was unable to evaluate cardiotoxicity over a long follow-up period. Finally, the study included various bone sarcomas, including osteosarcoma, cases of adult and older patients, and extremity and trunk sites. However, the results may be useful in demonstrating the clinical features of bone sarcoma of various subtypes, sites and age in actual practice.

In conclusion, the estimated five-year OS rate and necrosis rate following neoadjuvant and adjuvant chemotherapy with DXR and IFM for bone sarcomas in this study were acceptable in patients without metastasis prior to treatment. A better coordinated prospective study of this combination regimen for aged patients with bone sarcoma will be required to clarify its efficacy and tolerability.

## Figures and Tables

**Table I:** Baseline demographics and clinical characteristics.

Characteristic	Value
Gender, n (%)	
Male	12 (66)
Female	6 (34)
Histological subtype, n (%)	
Osteosarcoma	10 (56)
MFH of the bone	4 (22)
Dedifferentiated chondrosarcoma	3 (17)
Angiosarcoma of the bone	1 (6)
AJCC stage, n (%)	
IIA	4 (22)
IIB	10 (56)
IVA	1 (6)
IVB	3 (17)
Definitive treatment, n (%)	
Surgery	15 (83)
Heavy ion radiation	1 (6)
Surgery and heavy ion radiation	1 (6)
Inoperable with PD	1 (6)
Site of definitive treatment (n=17), n (%)	
Primary, extremity	9 (53)
Primary, trunk	5 (29)
Primary and metastasis	2 (12)
Solitary distant recurrence	1 (6)
Timing of chemotherapy, n (%)	
Neoadjuvant only	7 (39)
Adjuvant only	4 (22)
Neoadjuvant and adjuvant	7 (39)
Median age, years (range)	63 (28–76)
Median number of chemotherapy cycles (range)	4 (1–7)
Median follow up, months (range)	18.0 (4.6–73.7)

MFH, malignant fibrous histiocytoma; AJCC, American Joint Committee on Cancer; PD, progressive disease.

**Table II:** Efficacy data.

Response category	Value
RECIST response (n=14), n (%)	
SD	11 (79)
PD	3 (21)
Tissue response in SD and PD (n=12), n (%)	
Necrosis ≥90%	5 (42)
Necrosis <90%	7 (58)
Final status (n=18), n (%)	
NED	9 (50)
AWD	2 (11)
DOD	7 (39)
Estimated five-year OS rate, %	
AJCC stage II	56
AJCC stage IV	25

RECIST, response evaluation criteria in solid tumors; SD, stable disease; PD, progressive disease; NED, no evidence of disease; AWD, alive with disease; DOD, dead of disease; OS, overall survival, AJCC, American Joint Committee on Cancer.
